# Minimal clinically important difference (MCID) of the SCL-20 measure of depression severity in patients with cancer and major depression

**DOI:** 10.1186/1745-6215-16-S2-P54

**Published:** 2015-11-16

**Authors:** Bethan Copsey, Jane Walker, Susan Dutton, Rebecca Fisher, Michael Sharpe

**Affiliations:** 1 Oxford Clinical Trials Research Unit, Centre for Statistics in Medicine, Nuffield Department of Orthopaedics, Rheumatology and Musculoskeletal Sciences, University of Oxford, Oxford, UK; 2 Psychological Medicine Research, Department of Psychiatry, University of Oxford, Oxford, UK

## Aim

To estimate the minimal clinically important difference of the SCL-20 measure of depression severity in patients with cancer and major depression.

## Design

Secondary analysis of two randomised controlled trials comparing the effectiveness of a collaborative care intervention with that of usual care in reducing depression severity (measured using the SCL-20).

## Methods

Anchor-based methods were used comparing the SCL-20 to self-assessed depression improvement. Distribution-based methods were used to determine whether the important differences when calculated based on patient opinion aligned with the statistical properties of the SCL-20 measure.

## Results

Using anchor based methods, the estimates using between-group difference were 0.56 at 12 weeks and 0.73 at 24 weeks and the estimates using within-patient change were 0.74 at 12 weeks and 0.90 at 24 weeks. All of the above estimates of the MCID exceed the minimum detectable change of 0.31. However, the results from distribution based methods were not in agreement, with half a standard deviation being 0.29. Further exploration of the results is currently being undertaken examining the effects of utilising the varying methodology.

## Conclusions

A range of estimates for the MCID of the SCL-20 measure have been found. The estimates from the anchor-based approach are much higher than the target differences of approximately 0.3 previously used in clinical trials powered on the SCL-20, suggesting that the improvement regarded as meaningful by patients is much higher than expected.

